# Removal and Fouling Influence of Microplastics in Fertilizer Driven Forward Osmosis for Wastewater Reclamation

**DOI:** 10.3390/membranes11110845

**Published:** 2021-10-29

**Authors:** Ziyan Wang, Keqiang Liu, Ya Gao, Guanhua Li, Zhenyu Li, Quanfu Wang, Liwei Guo, Tong Liu, Mohammed A. Al-Namazi, Sheng Li

**Affiliations:** 1 Guangdong Key Laboratory of Membrane Materials and Separation Technologies, Guangzhou Institute of Advanced Technology, Chinese Academy of Sciences, Guangzhou 511458, China; ziyan_wang2015@163.com (Z.W.); 18255061212@163.com (Y.G.); liguanhua1995@126.com (G.L.); guoliwei815@126.com (L.G.); 2 College of Food Science and Engineering, Northwest A&F University, Xianyang 712199, China; lizhenyu@nwafu.edu.cn; 3 Water Conservancy Development Research Center, Taihu Basin Authority, Ministry of Water Resources, Shanghai 200080, China; liukeqiang@tba.gov.cn; 4 School of Marine Science and Technology, Harbin Institute of Technology, Weihai 264209, China; 5 National Engineering Research Center for Modernization of Extraction and Separation of Traditional Chinese Medicine (TCM), Guangzhou Hanfang Pharmaceutical Co., Ltd., Guangzhou 510240, China; 6 Nanjing Zhongpeng Environmental Technology Co., Ltd., Nanjing 210026, China; liutong_sea@126.com; 7 Desalination Technologies Research Institute (DTRI), Saline Water Conversion Corporation (SWCC), Al-Jubail 31951, Saudi Arabia; Malnamazi@swcc.gov.sa; 8 Qingdao Yonglixing Water Purification Technology Co., Ltd., Qingdao 266061, China

**Keywords:** microplastics, nano-scale plastics, forward osmosis, membrane fouling

## Abstract

Insufficient removal of microplastics (MPs) and nanoplastics (NPs) may exert negative effects on the environment and human health during wastewater reclamation. The fertilizer-driven forward osmosis (FDFO) is an emerging potential technology to generate high-quality water for irrigation of hydroponic systems. In this study, the removal of MPs/NPs by the FDFO process together with their impact on FDFO membrane fouling was investigated, due to FDFO’s low molecular weight cut-off and energy requirement by using fertilizer as draw solution. Plastic particles with two different sizes (100 nm and 1 μm) and extracellular polymers released by real wastewater bacteria were utilized as model compounds for FDFO performance comparison. Results show that FDFO membrane system could generate high-quality irrigation water with only fertilizer, completely removing extracellular polymers, MPs and NPs from wastewater. It was found that the MPs and NPs themselves do not cause a significant membrane fouling. Moreover, it could help to reduce the membrane fouling caused by extracellular substances. That is probably because MPs and NPs helped to form a loose and porous fouling layer. Therefore, the FDFO process could be a long-term stable (low fouling) process for the reclamation of wastewater with high-quality requirements.

## 1. Introduction

With the population increase, economic development and different consumption style, freshwater demand has increased dramatically in the past decades, leading to a clean water scarcity in the world. According to the World Water Development Report 2018, 47% of the world’s population lives in regions that face water scarcity for at least one month of the year [1]. By 2050, the number of people suffering this problem will rise by 10%, and thus nearly 60% of the world’s population will be facing water scarcity at least one month of the year [1].

Meantime, the discharge of wastewater in the world exceeds 2.2 trillion m^3^/year [2]. In China, as a rapid developing economy of the world, the total wastewater discharge volume was ~70 billion m^3^/year in 2017 [3]. If wastewater was properly treated, it can be used for agricultural landscape irrigation, industrial applications, car washing, toilet flushing, lawn watering, firefighting, and many other purposes [4]. It could be a substantial measure to release the pressure of freshwater demand.

For different usage purposes, wastewater reuse often requires a variety of in-depth treatments to remove contaminants, such as organic matter, inorganic salts, heavy metals, micro-pollutants, etc. [5,6,7]. Recently, microplastics (MPs) and nanoplastics (NPs) have become emerging concerns for the wastewater reclamation. According to the Organization for Economic Co-operation and Development (OECD), production of global plastic waste has been increasing since 2015, with more than 300 million tons flowing into the environment every year [8]. In 2018, the annual output of plastic products reached 335 million tons, while only less than 1% of that was biodegradable plastics and nearly one-tenth of it entered the water environment as fragments [9].

MPs and NPs are very resistant to decomposition and their sizes are within the diameter ranges of 1 to 5000 μm and less than 0.1 μm, respectively [10,11]. In recent years, MPs have already been found in some ocean organisms in several studies, and they threaten marine biodiversity. They can harm the blood, lymphatic system, digestive system and reproductive system when they are ingested by marine organisms [12]. In addition, the high effective surface area of NPs can lead to the fact that more toxic chemicals can be absorbed and released by them [13]. When these MPs and NPs are ingested by animals and plants, they can easily enter the food chain, and eventually end up in the bodies of human beings [14].

Unfortunately, MPs and NPs cannot be completely removed by traditional water treatment technologies. MPs have been reported to be removed by 99.5% in membrane bioreactor (MBR) system and 97% in oxidation ditch (OD) system, but the main size of MPs was >500 μm (40%) and 62.5–125 μm (29%) [15]. In the previous studies, the removal of MPs and NPs ≤ 1 μm in size is still not well understood, and these types of plastics are considered to be more dangerous due to the high surface area for toxic substance adsorption. Therefore, the removal of these MPs and NPs with small sizes via emerging technologies is one of the focuses of future wastewater reuse research.

Membrane separation technology has been widely applied in wastewater treatment. The low-pressure membrane processes of microfiltration (MF) and ultrafiltration (UF) are mainly used in the membrane bioreactor system [16]. Moreover, the high-pressure membrane processes of nanofiltration (NF) and reverse osmosis (RO) have been reported to applied in the deep treatment of wastewater treatment plant (WWTP) effluent for reclamation purposes [17].

Forward osmosis (FO) membrane technology is an emerging membrane process which utilizes the osmotic pressure difference across a selectively permeable membrane as the driving force for the transport of water molecules. Compared with pressure-driven membrane processes, the FO process features many advantages including higher removal of contaminates in wastewater compared to MF and UF due to its high molecular weight cut-off, low operational pressures compared to NF and RO, and relatively low membrane fouling propensity. The FO process requires a high salt concentration solution as draw solution to extract water from the wastewater [18,19].

Normally, the recovery of water from the diluted draw solution needs to be done by another membrane separation process such as reverse osmosis (RO), which is a highly energy-consuming [20]. However, the fertilizer-driven FO (FDFO) process has been reported in the past decade [21]. By using FDFO, Phuntsho and his colleagues have reported that reclaimed wastewater can be directly used for irrigation of hydroponic systems [19,22], avoiding the high energy consumption process for water recovery from diluted draw solution. The application of FDFO in wastewater reclamation for irrigation can ease the water demand of agriculture, which covers almost 70% of the freshwater demand of the world [1].

However, in the previous studies, the removal of MPs/NPs in the FDFO process has not been addressed. Considering the emerging concerns mentioned in the previous paragraphs, it is crucial to evaluate the removal of MPs/NPs in the FDFO system, targeting high-quality water generation from wastewater for hydroponic irrigation. Moreover, the impact of the presence of MPs/NPs and their interaction with real bacterial extracellular polymers in wastewater on membrane fouling is important for the long-term stable operation of the FDFO process, which has not been investigated before as well.

Therefore, this paper will evaluate the feasibility of using FDFO membrane technology to treat synthetic wastewater containing MPs and NPs for high-quality fertilizer solution production for irrigation purposes, and we also investigate the efficiency of the FO process on the removal of MPs and NPs in synthetic wastewater. Moreover, as membrane fouling is considered to be a major problem in membrane filtration technology applications, especially in the condition of reclaiming domestic wastewater containing a significant amount of organics, the formation of membrane fouling on a FO membrane surface and the impact of MPs and NPs on the FO membrane fouling were also investigated in this study.

## 2. Materials and Methods

### 2.1. MPs and NPs

Monodisperse green fluorescent microspheres (1.0% (*w*/*v*) with a size of 1 μm solution and 1.0% (*w*/*v*) monodisperse red fluorescent microsphere with a size of 100 nm solution (Shanghai Ziqibio Co., Ltd., Shanghai, China) were used as model compounds for MPs and NPs for the synthetic wastewater preparation.

### 2.2. Production of Bacterial Extracellular Material

To simulate the real domestic wastewater, bacterial extracellular materials were isolated and utilized to simulate the biopolymers, such as protein and polysaccharides, in wastewater [23]. The bacterial extracellular material was produced with the following procedure.

Wastewater bacteria *Shigella flexneri* strain 301 and *Escherichia fergusonii* ATCC 35469 were isolated from sampled real wastewater and cultivated for the extraction of extracellular material used in this study. LB broth solution was applied to cultivate wastewater bacteria, and the produced extracellular material during bacteria cultivation was separated from bacteria via centrifugation [23]. The broth solution after cultivation was centrifuged at 4750 rpm for 30 min and filtered by 0.45 μm membrane filters to separate bacteria and medium after incubation, and then the settled bacteria were discarded.

Afterwards, the supernatant solution was transferred to membrane dialysis bags with a molecular weight cut-off (MWCO) of 3500 Da (Spectrum Laboratories, Inc., Selangor, Malaysia) for a 10-day dialysis against Milli-Q water (Merck Millipore, Burlington, MA, USA). The Milli-Q water for dialysis was renewed daily to maintain the driving force. After dialysis, the composition and structure of bacterial extracellular material were analyzed with proton nuclear magnetic resonance (NMR) and liquid chromatography-organic carbon detection (LC-OCD, Haarlem, The Netherlands), and then stored in a reagent bottle for later usage.

### 2.3. Feed and Draw Solutions

#### 2.3.1. Feed Solution

The feed solution (FS) of FDFO process was prepared in three groups for experiments by adding different concentrations of isolated bacterial extracellular polymers and plastic model compounds in Milli-Q water as shown in Table 1.

The extracellular polymers in feed solution were synthesized by adding both the stored extracellular polymers isolated from *Shigella flexneri* strain 301 and *Escherichia fergusonii* ATCC 35469 to reach a total 5 mg/L concentration (2.5 mg/L from each bacterial polymer). Regarding the inorganic components present in wastewater, NH_4_Cl, NaHCO_3_, KH_2_PO_4_, MgCl_2_·6H_2_O and CaCl_2_·2H_2_O (Shanghai Aladdin, Shanghai, China) were utilized to simulate the inorganic composition in wastewater with concentrations shown in Table 2.

#### 2.3.2. Draw Solution

KCl (1 mol/L; Shanghai Aladdin, Shanghai, China) was prepared and applied as FDFO draw solution (DS) for all experiments.

### 2.4. Experimental Setup

**Figure 1:**
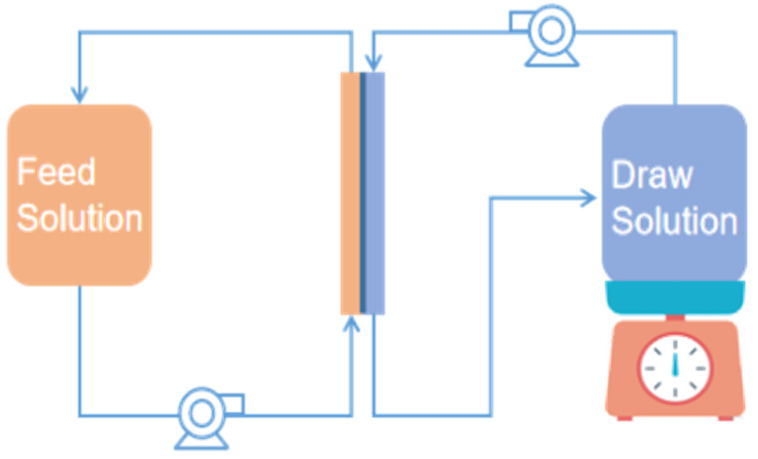
The flow chart of the FO membrane system.

As shown in Figure 1, experiments were conducted on a self-assembled forward osmosis membrane filtration system, which has been reported in a previous study [24]. The feed and draw solutions were separately recycled on both sides of FO membranes. Flat sheet cellulose triacetate FO membranes (HTI) with a 20 cm^2^ effective surface were used in this study. Detailed characteristics of this type of FO membrane can be found elsewhere [25].

### 2.5. Experimental Protocol

Before all experiments, the forward osmosis system was thoroughly cleaned by 200 ppm HCl and Milli-Q water to ensure that there was no bacteria or contaminant in this system. For each experiment, there were three cycles. Within each cycle, 1 L FS and 1 L DS were pumped into the FO system with a cross flow velocity of 8.5 m/s and circulated constantly. Newly prepared FS and DS were applied in each cycle of experiment.

During the experiment, extracted water from feed solution to draw solution was determined by measuring the real-time mass increase of draw solution via a digital balance with continuous data transfer to a computer. Then, the water fluxes were calculated via the Equation (1),

(1) $$F\;=\;\frac{\Delta m/\rho }{\Delta t\;\times \;A}$$

where F is the flux of FO process (L/m^2^/h), Δm is the increase of mass at a specific time period (g), ρ is the density of water, Δt is the time interval between two mass recording (h) and A is the surface area of the FO membrane used in the experiment (m^2^).

Besides the real-time measurement of FO process flux, sampling of FS and DS solutions was also performed before and after each cycle for the water quality analyses of protein, abundance of MPs and NPs, organic substance fraction concentrations via Bradford method, flow cytometer, fluorescence excitation-emission matrix (FEEM) and liquid chromatography-organic carbon detection (LC-OCD).

### 2.6. Analyses

#### 2.6.1. Protein

In this article, protein concentration was determined by Bradford method [26]. Bradford Dye Reagent (TaKaRa, Clontech, Otsu, Japan), Bovine serum albumin (BSA, Beyotime, Shanghai, China) and Biotek microplate reader (Elx808IU, Biotek, Winooski, VT, USA) were applied to detect protein.

#### 2.6.2. Flow Cytometer

The abundances of MPs and NPs were quantitatively determined by a flow cytometer (BD Accuri™ C6 Plus, BD Biosciences, San Jose, CA, USA) via the fluorescence detection.

#### 2.6.3. Scanning Electron Microscopy (SEM)

SEM was applied for observation of fouling layer on fouled membranes after experiments. Membrane samples were air-dried before imaging in SEM. Then, samples were sputter-coated with gold by carbon coater (SBC-12, KYKY, Beijing, China) and visualized on a Phenom XL desktop scanning electron microscope (Nanoscience Instruments, Phoenix, AZ, USA) at an accelerating voltage of 10kV.

#### 2.6.4. Proton Nuclear Magnetic Resonance (NMR)

^1^H nuclear magnetic resonance (NMR) analyses were conducted to understand the composition of extracellular substances released by bacteria in wastewater. The utilized NMR equipment, sample preparation and analysis protocols have been reported in the previous publication of the authors [23].

#### 2.6.5. Fluorescence Excitation-Emission Matrix (FEEM)

A fluorescence spectrometer (FluoroMAX-4 from Horiba, Kyoto, Japan) was used in this study. The FEEM measurements were performed using the identical settings and protocol applied in the previous studies [23]. Background signals were eliminated by subtracting the signals of the Milli-Q water (Merck Millipore, Burlington, MA, USA) from those of the samples.

To detect which fractions of isolated extracellular material are removed by FO system, two samples of each experiment were analyzed in FEEM: (1) feed solutions before FO filtration and (2) draw solutions after FO filtration.

#### 2.6.6. Liquid Chromatography-Organic Carbon Detection (LC-OCD)

A LC-OCD analysis instrument (DOC-LABOR Dr. Huber, Karlsruhe, Germany) was used in this study to characterize the different fractions of organic substances within the water samples as described by other researchers [27]. The principle of LC-OCD is size-exclusion chromatography with a combination of organic carbon and nitrogen detection. Before analysis, the samples were stored in a refrigerator at 4℃ until the measurements were conducted. The LC-OCD analysis can roughly provide information regarding the molecular weight distribution of organic material in the samples. Similar to the FEEM analyses, by analyzing the isolated extracellular material with LC-OCD before and after filtration in FO system, the efficiency of FO system on extracellular substances removal can be determined. Moreover, the SUVA and nitrogen/carbon (N/C) ratio can be determined for the different fractions of organics present in the water samples, indicating their corresponding properties [28].

## 3. Results

### 3.1. Characterization of Synthetic Wastewater

LC-OCD, FEEM and ^1^H NMR have been applied to characterize the organic composition of synthetic wastewater and evaluate the FDFO performance on removing those contaminants.

The principles of LC-OCD analyses are size exclusion, adsorption and elucidation. The higher molecular weights of organic substances are, the faster they could be eluted out for detection. In the LC-OCD chromatograms, biopolymers with high molecular weight (>20,000 Da) are normally the first portion of organics eluted out of the column (peak appearing at 30–35 min retention time), and then followed by humics and building blocks with a molecular weight around 1000 Da (peak appearing at 45–50 min retention time), and low molecular weight (LMW) neutrals and acids (<350 Da) appearing after 55 min retention time [27].

**Figure 2:**
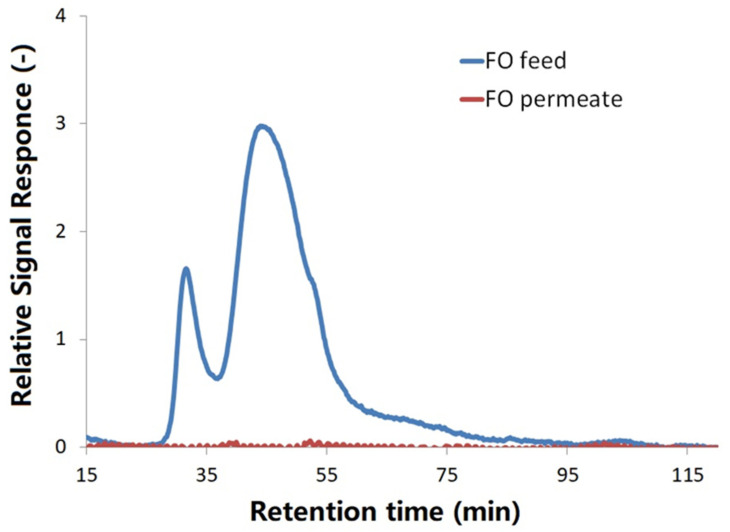
LC-OCD chromatograms of FDFO permeate (diluted draw solution) and initial feed solution with dissolved extracellular polymers released by two wastewater bacteria: *Shigella flexneri* strain 301 and *Escherichia fergusonii* ATCC 35469.

Figure 2 shows the LC-OCD chromatograms of FDFO permeate (red line) and the initial feed solution (blue line) with dissolved extracellular polymers released by two wastewater bacteria: *Shigella flexneri* strain 301 and *Escherichia fergusonii* ATCC 35469. As shown in Figure 2, the FO feed solution with added extracellular polymers from *Shigella* and *Escherichia* before the forward osmosis mainly exhibited two main peaks. According to the peaks assignment definitions of the LC-OCD manufacturer, the peak at 30–35 min retention time is biopolymer, which could be macro molecular weight organics, such as protein and polysaccharides, while the peak at about 45 min retention time indicating the presence of medium molecular weight humics and its breakdown building blocks. The chromatogram area that appears after 55 min retention time could be assigned to the presence of LMW neutrals [27].

**Figure 3:**
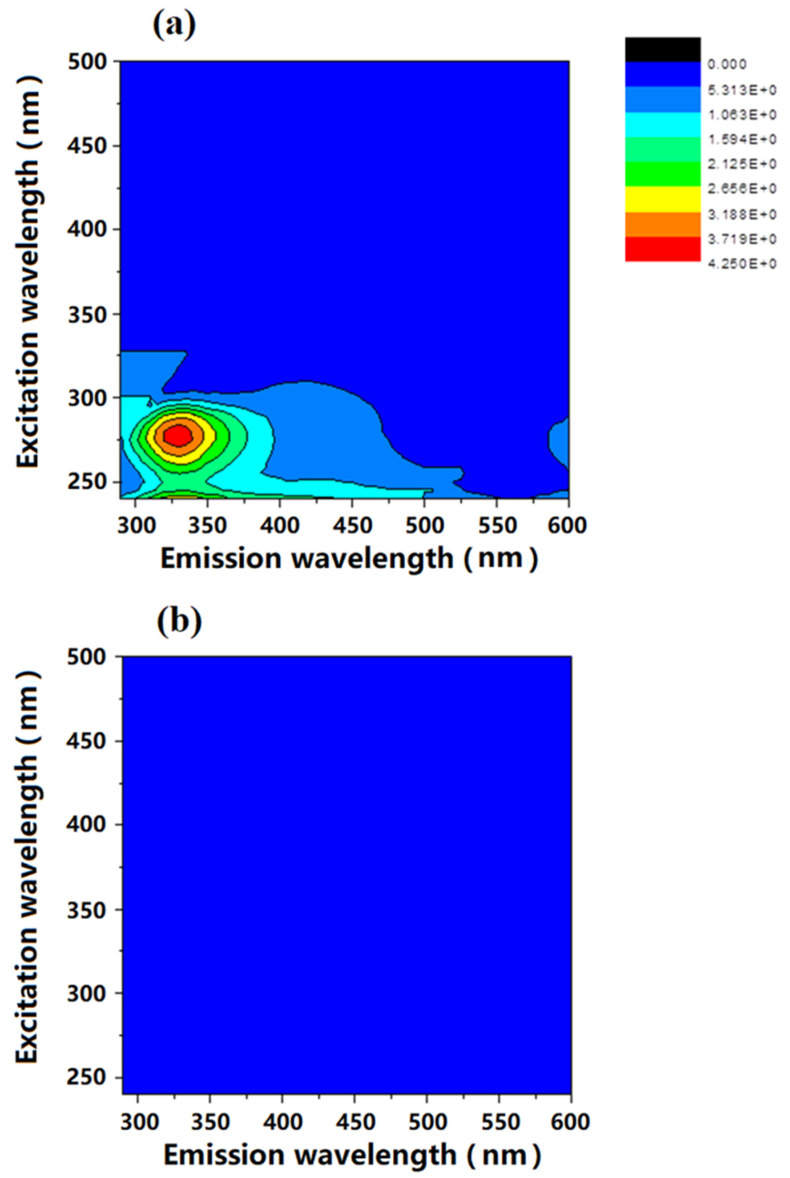
FEEM spectra of wastewater with isolated extracellular polymers from wastewater bacteria *Shigella flexneri* strain 301 and *Escherichia fergusonii* ATCC 35469 (**a**), and treated wastewater by the FDFO process (**b**).

The biopolymer in synthetic wastewater is mainly contributed by the added bacterial extracellular polymers. As the isolation in LC-OCD of biopolymers was based on size exclusion, the biopolymer could be protein and/or polysaccharide. To have a better understanding on the property of biopolymer fraction in synthetic wastewater, FEEM analysis was conducted in this study. Generally, acidic polysaccharides are considered to be one component of extracellular polymers. However, polysaccharides do not fluoresce; but humic-like and protein-like substances fluoresce. Therefore, the FEEM spectrum can provide an indication as to whether the biopolymer fraction of the synthetic wastewater contains proteins and humic substances. Figure 3 shows the FEEM spectra of synthetic wastewater with isolated extracellular material from wastewater bacteria and FO permeate (draw solution diluted by extracted water from feed) after removing extracellular polymers. As shown in Figure 3a, the FO feed solution with isolated extracellular polymers exhibits a main peak within the 310–350 nm emission and 260–280 nm excitation wavelength ranges in the FEEM spectra. Besides that, there is also a minor peak within 250–260 nm emission and 380–450 nm excitation wavelength ranges. According to the results of Villacorte and his colleagues [29], the main peak within the 310–350 nm emission and 260–280 nm excitation is usually considered to be protein-like substances, while the minor peak in the range of 250–260 nm emission and 380–450 nm excitation is humic-like substances.

By combining the analysis results obtained from LC-OCD and FEEM on the size distribution and chemical property, it was clear that the synthetic wastewater with real bacterial extracellular polymers contains macromolecular weight protein-like biopolymers and medium molecular weight humic substances.

**Figure 4:**
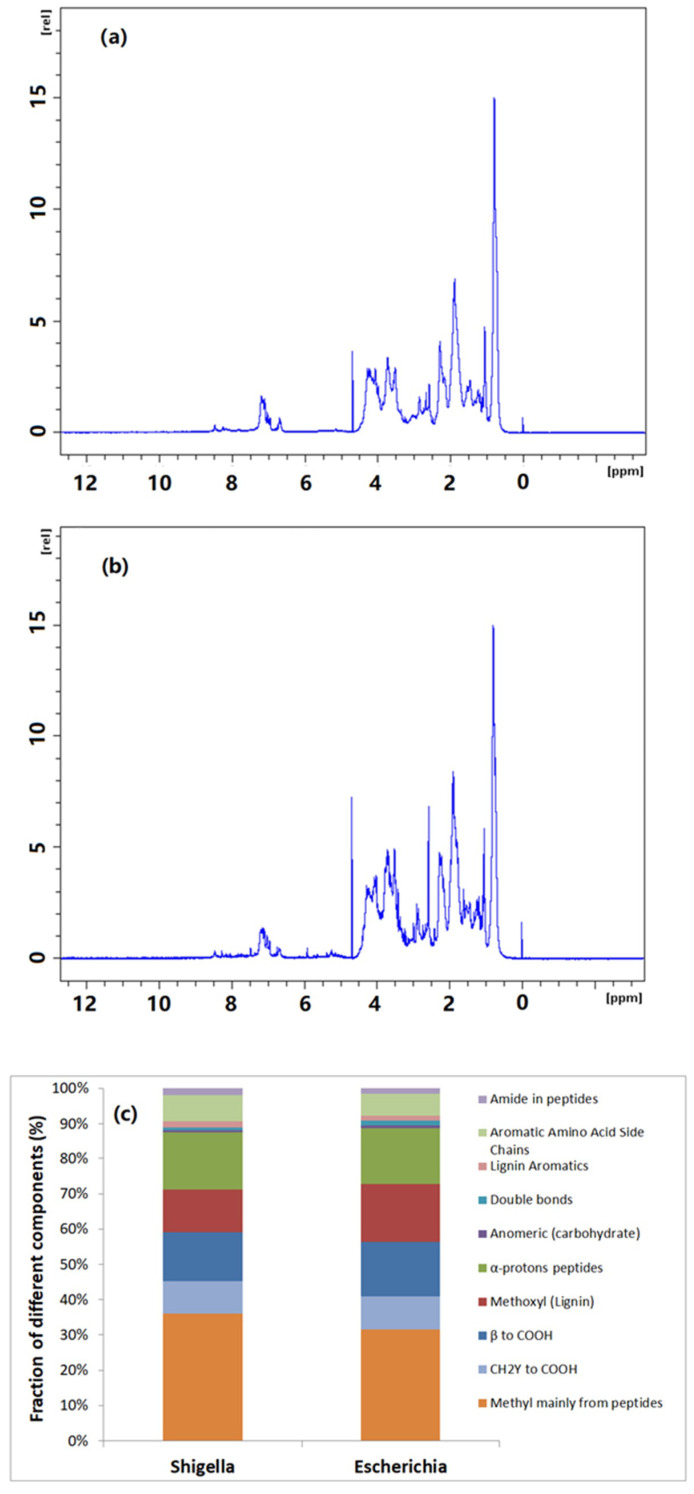
1H solution-state NMR chromatography at 700 MHz of isolated extracellular polymers from *Shigella flexneri* strain 301 (**a**), *Escherichia fergusonii* ATCC 35469 (**b**) and their corresponding fraction assignments and integration based on the NMR chromatography (**c**).

To further illustrate the structural composition of bacterial extracellular polymers in wastewater and understand the potential membrane foulants, proton solution-state NMR was conducted. The 1H solution-state NMR spectra at 700 MHz for bacterial extracellular polymers and the corresponding chemical shift assignment and interpretation of different peaks are shown in Figure 4. Although the chromatograms are not identical for the two isolated wastewater bacteria in terms of signal strength, the locations where peaks appear are similar, indicating a similar structural composition of the two bacterial extracellular polymers. The NMR chemical shift assignment and interpretation of different peaks was carried out based on the article published by Simpson and his colleagues [30]. Typically, aliphatic, carbohydrate and acrylamide/aromatic groups occur at the peaks of 0.7–2.3 ppm, 2.5–5.2 ppm and 6.4–8.5 ppm chemical shift, respectively. In the aliphatic population, peaks of 0.8–1.0 ppm usually represent the methyl group of peptides, which is credible evidence of the presence of proteins in the samples. On the other hand, peaks at 1.5 ppm and 2.2 ppm could be assigned to CH2γ to –COOH and β to –COOH, respectively. Peaks at 3.7 ppm, 4.3 ppm, 5.0 ppm and 5.3 ppm could be assigned to methoxyl (lignin), α-proton peptides, anomeric (carbohydrate) and double bonds, respectively. In terms of the amide/aromatic group, the peaks at 6.6 ppm, 7.1 ppm and 8.2 ppm were assigned to lignin aromatics, aromatic amino acid side chains and amide in peptides, respectively. As shown in Figure 4a,b, two bacterial extracellular polymers displayed clear signals in the ranges of 0.8–1.0 ppm and 7.0–7.5 ppm for methyl groups. Those are mainly from peptides and aromatic amino acid side chains, indicating the presence of protein substances in those samples.

The different detected compositional fractions in proton NMR analysis were quantitatively integrated according to their peak area in spectra as shown in Figure 4c. The methyl of peptides is the largest portion for both types of bacterial extracellular polymers (more than 30%). Considering other protein-like fractions, such as α-proton peptides (~20% of total organic substances), the protein-like substances was the main component within the bacterial extracellular polymers investigated in this study.

As mentioned in Section 2, commercial fluorescence plastic spheres were used in this study as MPs and NPs model compounds. Flow cytometry was applied for the quantification of MPs/NPs in FDFO feed solution and extracted water (draw solution after experiment). Table 3 shows the MPs and NPs in initial FO feed solution and draw solution after experiments. As shown in Table 3, 3–4 plastic particles in micro- and nanoscale were present per µL of FO feed solution.

### 3.2. Contaminants Removal by FDFO Process

The effectiveness of the FDFO process on removing contaminants in synthetic wastewater was evaluated by comparing the organics removal in LC-OCD and FEEM analyses, MPs/NPs removal in flow cytometry analysis.

As shown in Figure 3b, there is no peak observed in the FDFO-reclaimed water (draw solution after experiment) in both LC-OCD and FEEM spectra, which indicates all protein-like and humic-like organics were well removed by the FDFO process. Both results from LC-OCD and FEEM were consistent and indicated that the organic contaminants in synthetic wastewater can be comprehensively removed.

Moreover, because of the molecular weight cut-off of FO membranes (around 200 Da, comparable with RO membranes), there were no MPs and NPs detected by the flow cytometer in the FDFO reclaimed water as expected, indicating that a complete removal of plastics by the FDFO process. The good removal of MPs and NPs from wastewater could substantially reduce the risk of plastics associated with contaminants coming into human’s food web via wastewater reuse.

### 3.3. Membrane Fouling of the FDFO Process

To determine whether an emerging technique could be applicable in practice, the stability of long-term operation is crucial. Membrane fouling has been a main challenge in membrane filtration, and the impact of bacterial extracellular polymers and MPs/NPs on the FO membrane fouling was evaluated.

**Figure 5:**
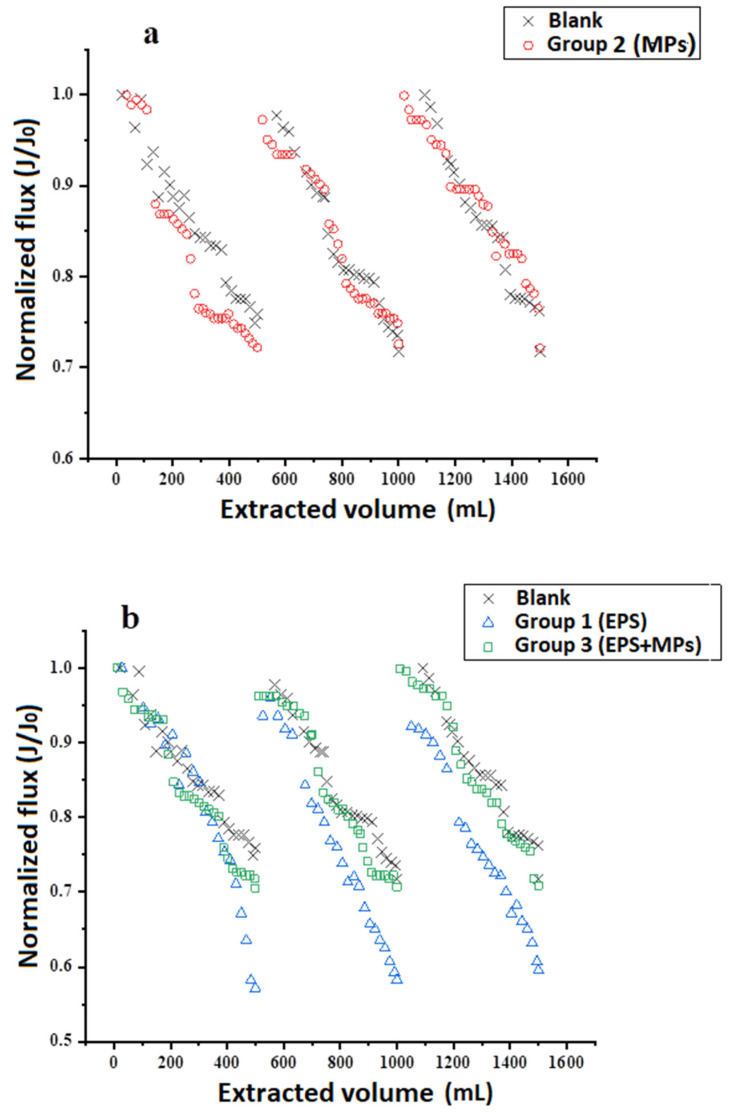
The comparison of normalized fluxes as a function of time between blank control and feed solution with only MPs and NPs (**a**), and between blank control, feed solution with extracellular polymers and feed solution with both extracellular polymers and plastics (**b**).

The normalized fluxes as a function of time between blank control and different feed solutions were compared in Figure 5. As shown in Figure 5a, the normalized flux of blank and feed solution with only MPs and NPs decreased from 1 to ~0.7 within one cycle for both cases. As the driving force of the FO process decreases with the concentration reduction of draw solution, which is caused by the dilution of water extracted from the feed solution, it is logical that the flux declined with the increase of extracted water in each cycle. If there no fouling occurred, the initial flux could be recovered when the feed and draw solutions were renewed at the next cycle. It was interesting to find that the feed solution with only MPs and NPs also exhibited a similar flux decline pattern as the blank control, indicating no fouling happened in the case of extracting water from synthetic wastewater containing only plastics. That was probably because the MPs and NPs are round-shape polymers. Although there were plastic particles deposited on the FO membrane surface, the porosity of the microspheres layer of plastic particles was high and would not enhance the resistance of membrane filtration.

However, as shown in Figure 5b, higher normalized flux reductions within each cycle were observed for the feed solution with bacterial extracellular polymers than blank (from 1 to around 0.57). Moreover, the reduction of initial flux of each cycle (~8%) for feed solution with only bacterial extracellular polymers was more severe than the blank, which exhibited almost no reduction (Figure 5), indicating the bacterial extracellular polymers in wastewater caused the FO membrane fouling due to their deposition of on the surface of FO membranes.

In contrast to the feed solution with only bacterial extracellular polymers, only a small decrease in membrane normalized flux was observed for the feed solution containing both bacterial extracellular polymers and plastic particles (Group 3). The presence of MPs and NPs in feed solution substantially reduced flux decline caused by the extracellular polymers, instead of enhancing the membrane fouling.

## 4. Discussion

The experimental results have proven that the FDFO process can generate high-quality reclaimed water from the wastewater by eliminating all the contaminants present in the wastewater.

Because of the good removal of both organic contaminants and plastics at micro- and nanoscale, and given the fact that the draw solution of FDFO system is fertilizer suitable for irrigation, the application of FDFO process for hydroponic systems would be promising. By removing all the organic contaminants in wastewater, the risk of up-taking the remaining pollutants in reclaimed wastewater in food web via reclaim wastewater irrigation could be substantially reduced. The additional advantage of the FDFO process is that the draw solute (fertilizer) can be specifically selected according to the requirements of specific crops cultivated in the hydroponic systems, which could maximize the production of hydroponic systems.

However, to determine whether an emerging technology can be applied in practice, the long-term stability is crucial. Similar to other membrane filtration process, membrane fouling is also the main challenge for the stability of FDFO process [19].

**Figure 6:**
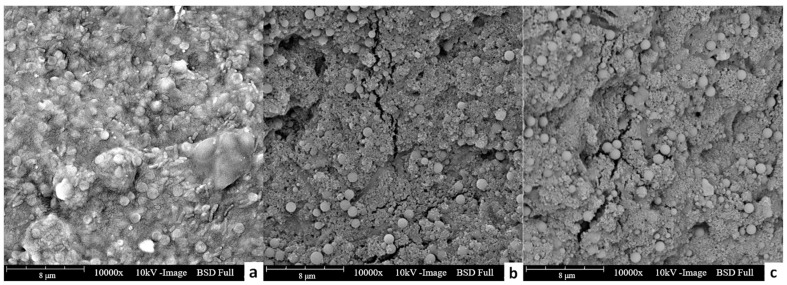
The SEM image of FO membranes after experiments treating feed solutions of in Group 1 (**a**), Group 2 (**b**) and Group 3 (**c**). The magnitude of these SEM images is 10,000×.

The serious FO fouling of treating feed solution with bacterial extracellular polymers was most likely caused by the accumulation of proteins in the synthetic wastewater. As the water in feed solution was extracted into draw solution during the process, the protein in the feed solution (main component of extracellular polymers, more than 50%) should be concentrated after the experiment if there was no deposition on membrane surface. However, the results in Table 4 show that the protein content in the feed solution after the experiment decreased for both cases treating extracellular polymers containing feed solutions, indicating the deposition of these substances on the membrane surface. The SEM image of fouled membranes after experiments also confirmed the deposition of extracellular polymers on membranes, which was consistent with the protein analyses (Figure 6). Moreover, the amount of protein reduction in Group 3 (feed solution with two kinds of microplastics and bacterial extracellular polymer) was smaller than that in Group 1 (feed solution with only bacterial extracellular polymer). This indicates that a smaller amount of protein was attached in Group 3, matching the lower normalized flux decline for the feed solution containing both bacterial extracellular polymers and plastic particles shown in Figure 5. The high fouling in feed solutions with bacterial extracellular polymers was probably because of the sticky nature and affinity of proteins in wastewater to the membrane surface. It has been reported that those substances are key fouling factors of membrane filtration [31].

On the other hand, the stickiness and compactness of the fouling layer might be reduced when the feed solution with MPs/NPs was treated together with bacterial extracellular polymers, and thus reduced the filtration resistance, resulting in a lower flux decline. Based on the SEM images of FO membranes treating different groups of feed solutions (Figure 6), it is clear that the fouling layer with plastic particles is a loose structure with many cracks, which is helpful for the water penetration. As shown in Figure 6a, a smooth and continuous fouling layer was formed on the surface of the forward osmosis membrane when feed solution with only extracellular polymers was treated. The continuous fouling layer completely blocked the contact between the feed solution and the membrane, and thus hindered the diffusion of water molecules through FO membranes, resulting in a significant reduction in membrane flux. On the contrary, a porous fouling layer with some tiny cracks was formed on the membrane surface when the MPs/NPs were present in the feed solutions. This porous structure of fouling layer could be easier to penetrate for water molecules, leading to a less membrane filtration resistance. Moreover, the loose fouling layer could be easily washed off by cleaning.

## 5. Conclusions

The FDFO process as an emerging technology was investigated in terms of its efficiency on the removal of organic contaminants and plastic particles in both micro- and nanoscale sizes. The following conclusions could be drawn based on the experimental results:

- Bacterial extracellular polymers are mainly composed of proteins, polysaccharides and humic substances. Protein-like substance comprise the main component of extracellular polymers, which covers more than 50% of the total organic substances.
- All tested contaminants in synthetic wastewater, including extracellular polymers, MPs and NPs, could be completely removed by the FDFO process, leading to a successful generation of high quality fertilizer solution without contaminants from domestic wastewater, which is important for relieving the freshwater stress.
- Serious FO fouling was observed when the synthetic wastewater with only bacterial extracellular polymers was treated (up to 8% normalized initial flux reduction). That was probably caused by the accumulation of proteins, polysaccharides and humics in wastewater forming a dense and compact fouling layer on the membrane surface, which enhanced the membrane filtration resistance.
- The MPs and NPs themselves do not cause membrane fouling (similar to the blank control experiment, almost no initial flux reduction). Moreover, when the MPs/NPs were present in the synthetic wastewater together with the bacterial extracellular polymers, it could reduce the membrane fouling caused by extracellular substances. That is probably because the fouling layer is a loose structure with the presence of MPs and NPs.

## Figures and Tables

**Table 1:** The composition of feed solutions in three groups in the FO system.

	Group 1	Group 2	Group 3	Blank
Extracellular polymers (mL/L)	5	0	5	0
Polystyrene of 1 μm (mg/L)	0	0.5	0.5	0
Polystyrene of 100 nm (mg/L)	0	0.5	0.5	0

**Table 2:** Inorganic composition of the synthetic wastewater used in this study.

Components	Value
NaHCO_3_ (mg/L)	100
KH_2_PO_4_ (mg/L)	20
NH_4_Cl (mg/L)	25
MgCl_2_·6H_2_O (mg/L)	10
CaCl_2_·2H_2_O (mg/L)	5

**Table 3:** Plastics present in feed and draw solutions of Group 2.

	Number of Plastics per µL		
	Measurement 1	Measurement 2	Measurement 3
Feed (before experiment)	3	4	4
Draw (after experiment)	N.D	N.D	N.D

Note: N.D stands for not detectable.

**Table 4:** The concentration of protein in FS in the FO system.

Sample	Average Concentration (μg/mL)
FS of Group 1 at beginning	14.94
FS of Group 1 in the end	11.30
FS of Group 3 at beginning	9.79
FS of Group 3 in the end	9.18

## Data Availability

The data presented in this study are available on request from the corresponding author. The data are not publicly available due to the restriction of research collaborator: Guangzhou Hanfang Pharmaceutical Co., Ltd.
